# *N*-(3-Eth­oxy­phen­yl)-4-methyl­benzene­sulfonamide

**DOI:** 10.1107/S1600536810022427

**Published:** 2010-06-16

**Authors:** Aziz-ur-Rehman, Asia Siddiqa, Mehmet Akkurt, Muhammad Athar Abbasi, Muhammad Jahangir, Islam Ullah Khan

**Affiliations:** a Department of Chemistry, Government College University, Lahore 54000, Pakistan; b Department of Physics, Faculty of Arts and Sciences, Erciyes University, 38039 Kayseri, Turkey

## Abstract

In the title compound, C_15_H_17_NO_3_S, the two aromatic rings make a dihedral angle of 69.42 (9)° with each other and the bridging C—N—S—C torsion angle is 65.76 (16)°. Weak intra­molecular C—H⋯O inter­actions may affect the mol­ecular conformation. Two neighbouring mol­ecules generate a hydrogen-bonded dimer about a center of inversion through a pair of inter­molecular N—H⋯O inter­actions, forming an *R*_2_^2^(8) ring motif. Furthermore, two inter­molecular C—H⋯π inter­actions contribute to the stability of the crystal packing.

## Related literature

For the biological activity of sulfonamides, see: Berredjem *et al.* (2000 ▶); Lee & Lee (2002 ▶); Soledade *et al.* (2006 ▶); Xiao & Timberlake (2000 ▶).
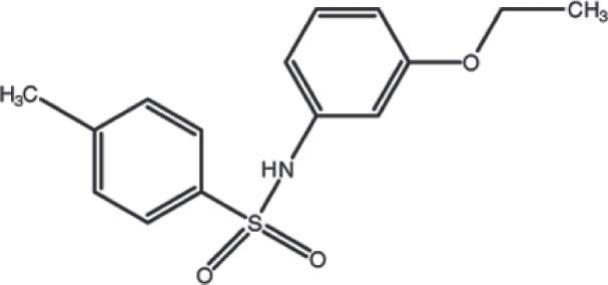


## Experimental

### 

#### Crystal data

C_15_H_17_NO_3_S
*M* *_r_* = 291.37
Monoclinic, 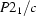

*a* = 8.4612 (3) Å
*b* = 13.1862 (5) Å
*c* = 13.4237 (4) Å
β = 99.326 (2)°
*V* = 1477.90 (9) Å^3^
*Z* = 4
Mo *K*α radiation
μ = 0.23 mm^−1^
*T* = 296 K
0.34 × 0.18 × 0.16 mm

#### Data collection

Bruker APEXII CCD diffractometer
13221 measured reflections
3608 independent reflections
2532 reflections with *I* > 2σ(*I*)
*R*_int_ = 0.036

#### Refinement

*R*[*F* ^2^ > 2σ(*F* ^2^)] = 0.043
*wR*(*F* ^2^) = 0.118
*S* = 1.00
3608 reflections
186 parameters
1 restraint
H atoms treated by a mixture of independent and constrained refinement
Δρ_max_ = 0.30 e Å^−3^
Δρ_min_ = −0.31 e Å^−3^

### 

Data collection: *APEX2* (Bruker, 2007 ▶); cell refinement: *SAINT* (Bruker, 2007 ▶); data reduction: *SAINT*; program(s) used to solve structure: *SHELXS97* (Sheldrick, 2008 ▶); program(s) used to refine structure: *SHELXL97* (Sheldrick, 2008 ▶); molecular graphics: *ORTEP-3 for Windows* (Farrugia, 1997 ▶); software used to prepare material for publication: *WinGX* (Farrugia, 1999 ▶) and *PLATON* (Spek, 2009 ▶).

## Supplementary Material

Crystal structure: contains datablocks global, I. DOI: 10.1107/S1600536810022427/sj5019sup1.cif

Structure factors: contains datablocks I. DOI: 10.1107/S1600536810022427/sj5019Isup2.hkl

Additional supplementary materials:  crystallographic information; 3D view; checkCIF report

## Figures and Tables

**Table 1:** Hydrogen-bond geometry (Å, °) *Cg*1 and *Cg*2 are the centroids of the C2–C7 and C8–C13 benzene rings, respectively.

*D*—H⋯*A*	*D*—H	H⋯*A*	*D*⋯*A*	*D*—H⋯*A*
N1—H1*N*⋯O2^i^	0.821 (16)	2.140 (17)	2.9476 (19)	167.9 (16)
C4—H4⋯O2	0.93	2.54	2.914 (2)	104
C13—H13⋯O1	0.93	2.42	3.019 (2)	122
C14—H14*A*⋯*Cg*1^ii^	0.97	2.90	3.752 (3)	147
C15—H15*C*⋯*Cg*2^iii^	0.96	2.96	3.763 (3)	147

Symmetry codes: (i) 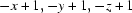; (ii) 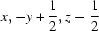; (iii) 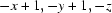.

**Fig. 1.**
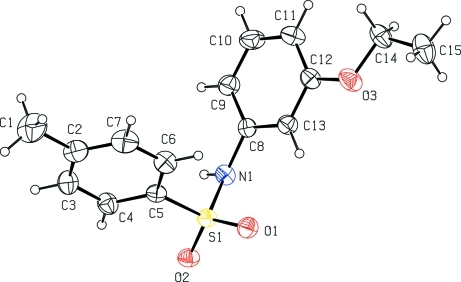
The title molecule of (I), with displacement ellipsoids drawn at the 30% probability level.

**Fig. 2.**
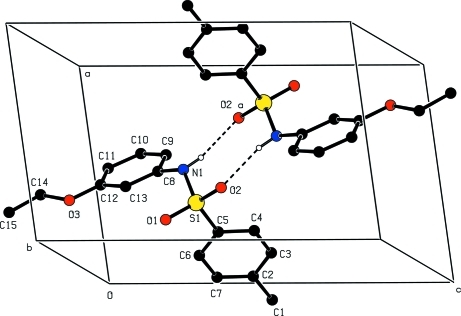
View of the N—H···O dimer in the unit cell of (I). H-atoms not involved in hydrogen bonds have been omitted for clarity.

## supplementary crystallographic information

### Comment

Sulfonamide is an important functionality found in a number of synthetic as well as natural compounds possessing versatile type of biological activities *e.g.* herbicidal, anti-malarial, anti-convulsant and anti-hypertensive (Soledade *et al.*, 2006; Xiao & Timberlake, 2000; Berredjem *et al.*, 2000; Lee & Lee, 2002) activities. In the present paper, the structure of *N*-(3-ethoxyphenyl)-4-methylbenzene sulfonamide has been determined as part of a research program involving the synthesis and biological evaluation of sulfur containing compounds.

In the title compound (I), Fig. 1), the dihedral angle between the two aromatic rings (C2–C7 and C8–C13) is 69.42 (9)° and the bridging C5—S1—N1—C8 torsion angle is 65.76 (16)°. In the crystal structure, two neighbouring molecules generate a hydrogen-bonded dimer about a center of inversion through a pair of intermolecular N—H···O interactions, forming an *R*_2_^2^(8) ring motif (Table 1, Fig. 2).

In the structure, two intermolecular C—H···π interactions contribute to the stability of crystal packing (Table 1).

### Experimental

A mixture of 4-methyl benzene sulfonyl chloride (10.0 mmoles; 1.90 g), 3-ethoxy aniline (*meta*-phenetidine) (10.0 mmoles; 1.25 g), aqueous sodium carbonate (10%; 10.0 ml) and water (25 ml) was stirred for half an hour at room temperature. The crude mixture was washed with water and dried. Product was dissolved in methanol and crystallized by slow evaporation of the solvent. Yield 72%. 4-Methyl benzene sulfonyl chloride and *meta*-phenetidine were purchased from Sigma Aldrich while all other chemicals involved were obtained from Merk, Germany.

### Refinement

H atoms bonded to C atoms were positioned geometrically and refined using a riding model, with C—H = 0.93–0.97Å and *U*_iso_(H) = 1.2–1.5*U*_eq_ (C). The amino H-atom was found in a difference Fourier map, and refined with a distance restraint of N–H 0.86 (2) Å. The H-atom *U*_iso_ parameter was fixed at 1.2*U*_eq_(N) for the N—H group.

### Crystal data

**Table:**

C_15_H_17_NO_3_S	*F*(000) = 616
*M**_r_* = 291.37	*D*_x_ = 1.309 Mg m^−^^3^
Monoclinic, *P*2_1_/*c*	Mo *K*α radiation, λ = 0.71073 Å
Hall symbol: -P 2ybc	Cell parameters from 4223 reflections
*a* = 8.4612 (3) Å	θ = 2.9–26.1°
*b* = 13.1862 (5) Å	µ = 0.23 mm^−^^1^
*c* = 13.4237 (4) Å	*T* = 296 K
β = 99.326 (2)°	Needle, colourless
*V* = 1477.90 (9) Å^3^	0.34 × 0.18 × 0.16 mm
*Z* = 4	

### Data collection

**Table:**

Bruker APEXII CCD diffractometer	2532 reflections with *I* > 2σ(*I*)
Radiation source: sealed tube	*R*_int_ = 0.036
graphite	θ_max_ = 28.3°, θ_min_ = 3.4°
φ and ω scans	*h* = −11→11
13221 measured reflections	*k* = −17→15
3608 independent reflections	*l* = −17→17

### Refinement

**Table:**

Refinement on *F*^2^	Primary atom site location: structure-invariant direct methods
Least-squares matrix: full	Secondary atom site location: difference Fourier map
*R*[*F*^2^ > 2σ(*F*^2^)] = 0.043	Hydrogen site location: inferred from neighbouring sites
*wR*(*F*^2^) = 0.118	H atoms treated by a mixture of independent and constrained refinement
*S* = 1.00	*w* = 1/[σ^2^(*F*_o_^2^) + (0.0564*P*)^2^ + 0.2791*P*] where *P* = (*F*_o_^2^ + 2*F*_c_^2^)/3
3608 reflections	(Δ/σ)_max_ < 0.001
186 parameters	Δρ_max_ = 0.30 e Å^−^^3^
1 restraint	Δρ_min_ = −0.31 e Å^−^^3^

### Special details

**Table:**

Geometry. Bond distances, angles *etc*. have been calculated using the rounded fractional coordinates. All su's are estimated from the variances of the (full) variance-covariance matrix. The cell e.s.d.'s are taken into account in the estimation of distances, angles and torsion angles
Refinement. Refinement on *F*^2^ for ALL reflections except those flagged by the user for potential systematic errors. Weighted *R*-factors *wR* and all goodnesses of fit *S* are based on *F*^2^, conventional *R*-factors *R* are based on *F*, with *F* set to zero for negative *F*^2^. The observed criterion of *F*^2^ > σ(*F*^2^) is used only for calculating -*R*-factor-obs *etc*. and is not relevant to the choice of reflections for refinement. *R*-factors based on *F*^2^ are statistically about twice as large as those based on *F*, and *R*-factors based on ALL data will be even larger.

### Fractional atomic coordinates and isotropic or equivalent isotropic displacement parameters (Å^2^)

**Table:**

	*x*	*y*	*z*	*U*_iso_*/*U*_eq_	
S1	0.26616 (5)	0.52544 (3)	0.38686 (3)	0.0408 (1)	
O1	0.17864 (15)	0.57574 (10)	0.30139 (9)	0.0518 (4)	
O2	0.32376 (14)	0.58201 (9)	0.47639 (9)	0.0495 (4)	
O3	0.30011 (17)	0.42300 (12)	−0.00035 (9)	0.0653 (5)	
N1	0.42808 (16)	0.47848 (12)	0.35383 (11)	0.0439 (5)	
C1	−0.1104 (3)	0.1674 (2)	0.4986 (2)	0.1033 (13)	
C2	−0.0197 (2)	0.25798 (16)	0.47044 (18)	0.0623 (7)	
C3	0.0767 (2)	0.31285 (17)	0.54323 (16)	0.0639 (8)	
C4	0.1627 (2)	0.39529 (15)	0.51876 (14)	0.0532 (6)	
C5	0.15151 (18)	0.42365 (13)	0.41926 (13)	0.0414 (5)	
C6	0.0547 (2)	0.37042 (16)	0.34460 (15)	0.0562 (7)	
C7	−0.0295 (2)	0.28784 (18)	0.37117 (18)	0.0673 (8)	
C8	0.43243 (18)	0.41644 (13)	0.26721 (12)	0.0402 (5)	
C9	0.5253 (2)	0.32988 (15)	0.28030 (15)	0.0548 (6)	
C10	0.5428 (3)	0.27331 (17)	0.19682 (16)	0.0668 (8)	
C11	0.4705 (2)	0.30085 (16)	0.10134 (15)	0.0591 (7)	
C12	0.3773 (2)	0.38714 (15)	0.08949 (13)	0.0484 (6)	
C13	0.3581 (2)	0.44493 (14)	0.17269 (13)	0.0466 (5)	
C14	0.3347 (3)	0.37772 (18)	−0.09048 (13)	0.0624 (7)	
C15	0.2431 (3)	0.4353 (2)	−0.17758 (16)	0.0836 (9)	
H1A	−0.20100	0.15520	0.44690	0.1550*	
H1B	−0.14680	0.18010	0.56160	0.1550*	
H1C	−0.04160	0.10910	0.50520	0.1550*	
H1N	0.486 (2)	0.4612 (14)	0.4062 (12)	0.0530*	
H3	0.08400	0.29390	0.61060	0.0770*	
H4	0.22780	0.43140	0.56900	0.0640*	
H6	0.04630	0.38990	0.27740	0.0670*	
H7	−0.09440	0.25150	0.32100	0.0810*	
H9	0.57500	0.31030	0.34430	0.0660*	
H10	0.60510	0.21490	0.20500	0.0800*	
H11	0.48420	0.26180	0.04570	0.0710*	
H13	0.29490	0.50300	0.16470	0.0560*	
H14A	0.30240	0.30710	−0.09370	0.0750*	
H14B	0.44870	0.38110	−0.09240	0.0750*	
H15A	0.13040	0.42930	−0.17630	0.1250*	
H15B	0.26700	0.40800	−0.23980	0.1250*	
H15C	0.27350	0.50550	−0.17240	0.1250*	

### Atomic displacement parameters (Å^2^)

**Table:**

	*U*^11^	*U*^22^	*U*^33^	*U*^12^	*U*^13^	*U*^23^
S1	0.0391 (2)	0.0417 (2)	0.0397 (2)	0.0033 (2)	0.0004 (2)	−0.0018 (2)
O1	0.0518 (7)	0.0531 (8)	0.0479 (7)	0.0098 (6)	0.0001 (6)	0.0050 (6)
O2	0.0518 (7)	0.0455 (7)	0.0489 (7)	0.0015 (6)	0.0017 (5)	−0.0086 (5)
O3	0.0699 (9)	0.0826 (11)	0.0410 (7)	0.0166 (8)	0.0018 (6)	−0.0055 (7)
N1	0.0358 (7)	0.0540 (9)	0.0399 (8)	0.0022 (7)	0.0003 (6)	−0.0013 (7)
C1	0.0842 (17)	0.085 (2)	0.149 (3)	−0.0305 (15)	0.0439 (18)	−0.0015 (18)
C2	0.0435 (10)	0.0584 (13)	0.0886 (15)	−0.0062 (9)	0.0214 (10)	−0.0050 (11)
C3	0.0656 (12)	0.0657 (14)	0.0642 (13)	−0.0056 (11)	0.0218 (10)	0.0055 (10)
C4	0.0529 (10)	0.0608 (12)	0.0452 (10)	−0.0080 (9)	0.0060 (8)	−0.0050 (8)
C5	0.0345 (8)	0.0430 (10)	0.0457 (9)	0.0040 (7)	0.0037 (7)	−0.0041 (7)
C6	0.0475 (10)	0.0644 (13)	0.0532 (11)	−0.0058 (9)	−0.0022 (8)	−0.0056 (9)
C7	0.0462 (11)	0.0712 (15)	0.0816 (15)	−0.0128 (10)	0.0013 (10)	−0.0184 (12)
C8	0.0346 (8)	0.0438 (10)	0.0425 (9)	−0.0024 (7)	0.0069 (7)	0.0006 (7)
C9	0.0572 (11)	0.0536 (12)	0.0514 (10)	0.0122 (9)	0.0018 (9)	0.0024 (9)
C10	0.0728 (13)	0.0579 (13)	0.0669 (13)	0.0228 (11)	0.0030 (11)	−0.0065 (10)
C11	0.0590 (11)	0.0627 (13)	0.0549 (11)	0.0076 (10)	0.0076 (9)	−0.0155 (9)
C12	0.0431 (9)	0.0583 (11)	0.0428 (9)	0.0007 (8)	0.0044 (7)	−0.0032 (8)
C13	0.0441 (9)	0.0489 (10)	0.0459 (9)	0.0067 (8)	0.0049 (7)	0.0001 (8)
C14	0.0626 (12)	0.0828 (15)	0.0420 (10)	−0.0107 (11)	0.0092 (9)	−0.0129 (10)
C15	0.0858 (16)	0.116 (2)	0.0468 (12)	−0.0155 (16)	0.0038 (11)	0.0010 (13)

### Geometric parameters (Å, °)

**Table:**

S1—O1	1.4245 (13)	C11—C12	1.379 (3)
S1—O2	1.4313 (13)	C12—C13	1.383 (3)
S1—N1	1.6291 (15)	C14—C15	1.500 (3)
S1—C5	1.7517 (17)	C1—H1A	0.9600
O3—C12	1.360 (2)	C1—H1B	0.9600
O3—C14	1.422 (2)	C1—H1C	0.9600
N1—C8	1.427 (2)	C3—H3	0.9300
N1—H1N	0.821 (16)	C4—H4	0.9300
C1—C2	1.501 (3)	C6—H6	0.9300
C2—C7	1.379 (3)	C7—H7	0.9300
C2—C3	1.373 (3)	C9—H9	0.9300
C3—C4	1.377 (3)	C10—H10	0.9300
C4—C5	1.375 (3)	C11—H11	0.9300
C5—C6	1.379 (3)	C13—H13	0.9300
C6—C7	1.379 (3)	C14—H14A	0.9700
C8—C13	1.374 (2)	C14—H14B	0.9700
C8—C9	1.381 (3)	C15—H15A	0.9600
C9—C10	1.374 (3)	C15—H15B	0.9600
C10—C11	1.377 (3)	C15—H15C	0.9600
			
S1···H13	3.0500	H1N···O2^ii^	2.140 (17)
S1···H10^i^	3.0600	H3···H1B	2.4700
O1···C13	3.019 (2)	H4···O2	2.5400
O2···N1^ii^	2.9476 (19)	H4···H15B^vii^	2.5500
O1···H13	2.4200	H6···O1	2.6900
O1···H7^iii^	2.8600	H7···H1A	2.4000
O1···H10^i^	2.6000	H7···O1^vi^	2.8600
O1···H15A^iv^	2.8700	H9···H1N	2.3300
O1···H6	2.6900	H9···O2^ii^	2.8100
O2···H4	2.5400	H10···S1^viii^	3.0600
O2···H11^i^	2.9200	H10···O1^viii^	2.6000
O2···H1N^ii^	2.140 (17)	H11···C14	2.5600
O2···H9^ii^	2.8100	H11···H14A	2.3000
N1···O2^ii^	2.9476 (19)	H11···H14B	2.4100
C6···C8	3.569 (2)	H11···O2^viii^	2.9200
C8···C6	3.569 (2)	H13···S1	3.0500
C13···O1	3.019 (2)	H13···O1	2.4200
C7···H14A^v^	3.0400	H13···H1A^iii^	2.5500
C11···H14A	2.7700	H14A···C11	2.7700
C11···H14B	2.7900	H14A···H11	2.3000
C14···H11	2.5600	H14A···C7^ix^	3.0400
H1A···H7	2.4000	H14B···C11	2.7900
H1A···H13^vi^	2.5500	H14B···H11	2.4100
H1B···H3	2.4700	H15A···O1^iv^	2.8700
H1N···H9	2.3300	H15B···H4^x^	2.5500
			
O1—S1—O2	119.70 (8)	C2—C1—H1B	109.00
O1—S1—N1	107.95 (8)	C2—C1—H1C	109.00
O1—S1—C5	108.68 (8)	H1A—C1—H1B	109.00
O2—S1—N1	103.87 (7)	H1A—C1—H1C	109.00
O2—S1—C5	108.53 (8)	H1B—C1—H1C	110.00
N1—S1—C5	107.48 (8)	C2—C3—H3	119.00
C12—O3—C14	118.24 (16)	C4—C3—H3	119.00
S1—N1—C8	125.00 (11)	C3—C4—H4	120.00
C8—N1—H1N	116.6 (12)	C5—C4—H4	120.00
S1—N1—H1N	106.5 (12)	C5—C6—H6	121.00
C1—C2—C7	121.2 (2)	C7—C6—H6	121.00
C3—C2—C7	118.23 (19)	C2—C7—H7	119.00
C1—C2—C3	120.6 (2)	C6—C7—H7	119.00
C2—C3—C4	121.5 (2)	C8—C9—H9	121.00
C3—C4—C5	119.36 (17)	C10—C9—H9	121.00
S1—C5—C4	119.72 (13)	C9—C10—H10	119.00
C4—C5—C6	120.44 (17)	C11—C10—H10	119.00
S1—C5—C6	119.81 (14)	C10—C11—H11	120.00
C5—C6—C7	118.99 (19)	C12—C11—H11	121.00
C2—C7—C6	121.5 (2)	C8—C13—H13	120.00
N1—C8—C9	117.35 (15)	C12—C13—H13	120.00
N1—C8—C13	121.82 (15)	O3—C14—H14A	110.00
C9—C8—C13	120.63 (16)	O3—C14—H14B	110.00
C8—C9—C10	118.64 (18)	C15—C14—H14A	110.00
C9—C10—C11	121.7 (2)	C15—C14—H14B	110.00
C10—C11—C12	118.99 (19)	H14A—C14—H14B	108.00
O3—C12—C13	114.97 (17)	C14—C15—H15A	109.00
O3—C12—C11	124.94 (17)	C14—C15—H15B	109.00
C11—C12—C13	120.09 (17)	C14—C15—H15C	109.00
C8—C13—C12	119.93 (17)	H15A—C15—H15B	110.00
O3—C14—C15	107.41 (19)	H15A—C15—H15C	109.00
C2—C1—H1A	109.00	H15B—C15—H15C	109.00
			
O1—S1—N1—C8	−51.29 (16)	C1—C2—C3—C4	179.04 (19)
O2—S1—N1—C8	−179.35 (14)	C2—C3—C4—C5	0.2 (3)
C5—S1—N1—C8	65.76 (16)	C3—C4—C5—C6	0.3 (3)
O1—S1—C5—C4	−147.64 (14)	C3—C4—C5—S1	−177.61 (14)
O2—S1—C5—C4	−15.96 (16)	C4—C5—C6—C7	−0.6 (3)
N1—S1—C5—C4	95.79 (15)	S1—C5—C6—C7	177.28 (14)
O1—S1—C5—C6	34.49 (16)	C5—C6—C7—C2	0.4 (3)
O2—S1—C5—C6	166.16 (14)	N1—C8—C9—C10	−174.51 (18)
N1—S1—C5—C6	−82.09 (15)	C9—C8—C13—C12	−0.6 (3)
C14—O3—C12—C13	169.88 (18)	N1—C8—C13—C12	174.16 (16)
C12—O3—C14—C15	−176.27 (18)	C13—C8—C9—C10	0.5 (3)
C14—O3—C12—C11	−9.5 (3)	C8—C9—C10—C11	0.0 (3)
S1—N1—C8—C9	−133.67 (15)	C9—C10—C11—C12	−0.4 (3)
S1—N1—C8—C13	51.4 (2)	C10—C11—C12—C13	0.3 (3)
C3—C2—C7—C6	0.1 (3)	C10—C11—C12—O3	179.66 (19)
C1—C2—C7—C6	−179.4 (2)	O3—C12—C13—C8	−179.21 (16)
C7—C2—C3—C4	−0.4 (3)	C11—C12—C13—C8	0.2 (3)

Symmetry codes: (i) −*x*+1, *y*+1/2, −*z*+1/2; (ii) −*x*+1, −*y*+1, −*z*+1; (iii) −*x*, *y*+1/2, −*z*+1/2; (iv) −*x*, −*y*+1, −*z*; (v) *x*, −*y*+1/2, *z*+1/2; (vi) −*x*, *y*−1/2, −*z*+1/2; (vii) *x*, *y*, *z*+1; (viii) −*x*+1, *y*−1/2, −*z*+1/2; (ix) *x*, −*y*+1/2, *z*−1/2; (x) *x*, *y*, *z*−1.

### Hydrogen-bond geometry (Å, °)

**Table:**

Cg1 and Cg2 are the centroids of the C2–C7 and C8–C13 benzene rings, respectively.

**Table:**

*D*—H···*A*	*D*—H	H···*A*	*D*···*A*	*D*—H···*A*
N1—H1N···O2^ii^	0.821 (16)	2.140 (17)	2.9476 (19)	167.9 (16)
C4—H4···O2	0.93	2.54	2.914 (2)	104
C13—H13···O1	0.93	2.42	3.019 (2)	122
C14—H14A···Cg1^ix^	0.97	2.90	3.752 (3)	147
C15—H15C···Cg2^xi^	0.96	2.96	3.763 (3)	147

Symmetry codes: (ii) −*x*+1, −*y*+1, −*z*+1; (ix) *x*, −*y*+1/2, *z*−1/2; (xi) −*x*+1, −*y*+1, −*z*.
